# Pollen morphology and variability of invasive *Spiraea tomentosa* L. (Rosaceae) from populations in Poland

**DOI:** 10.1371/journal.pone.0218276

**Published:** 2019-08-23

**Authors:** Dorota Wrońska-Pilarek, Blanka Wiatrowska, Jan Bocianowski

**Affiliations:** 1 Department of Forest Botany, Poznan University of Life Sciences, Poznań, Poland; 2 Department of Mathematical and Statistical Methods, Poznan University of Life Sciences, Poznań, Poland; Kansas State University, UNITED STATES

## Abstract

The aim of this study was to investigate the pollen morphology and the ranges of intraspecific and interindividual variability of the North American steeplebush—*Spiraea tomentosa* L., an invasive species in Poland. Steeplebush inflorescences were collected randomly from 30 localities of *S*. *tomentosa* in Poland. In total, 900 pollen grains were analysed with both a light and a scanning electron microscope. Nine quantitative and three qualitative pollen features were studied. The diagnostic features were: exine ornamentation (size and direction of the muri), operculum and perforation size. For the first time, the intraspecific and interindividual variability of the pollen grains of the highly invasive *S*. *tomentosa* were investigated. Pollen grain features were so similar, that they did not allow to differentiate individual samples of *S*. *tomentosa* and only groups of samples were recognized.

## Introduction

The genus *Spiraea* L. belongs to the *Rosaceae* Juss. family, to the Amygdaloideae subfamily and to the Spiraeeae tribe, which is the subject of considerable speculation regarding its internal divisions. Recent phylogenetic analyses divided Spiraeeae into eight genera: *Aruncus*, *Holodiscus*, *Kelseya*, *Luetkea*, *Pentactina*, *Petrophyton*, *Sibiraea* and *Spiraea* [1, 2], although Flora of North America [3] included the *Xerospiraea* genus instead of the *Pentactina*.

Poyarkova [4] developed a system for the genus *Spiraea* s.l. subdivided into three sections: *Spiraria*, *Chamaedryon*, and *Calospira*. This genus is by far the largest and most widespread taxonomic unit in the tribe and includes c.a. 100–120 species [3], which grow in the Northern Hemisphere [5]. Hybridization occurs in natural settings, complicating identification of the species and varieties, and numerous horticultural hybrids have been created [3]. *Spiraea* is a very popular decorative plant in North America, Asia and in Europe. In the latter, many species of steeplebush from North America are cultivated, and some of them, for example, *S*. *tomentosa* or *S*. *douglasii*, have become established and invasive in some European countries [6], posing a threat to biodiversity in these areas [7].

*Spiraea tomentosa* L. naturally occurs in North America, where its range extends along the east coast, from Québec in Canada in the northern part of the cold temperate zone to Louisiana and Georgia, in a warm subtropical climate zone in the southern United States [8]. Scattered positions have also been found in Washington and Oregon, states located along thecoast of the Pacific Ocean. *S*. *tomentosa* was cultivated as early as the first half of the eighteenth century [9]. Currently, it is recognized in eight European countries as a fully acclimated neophyte [6, 10], and is considered an invasive species in four countries: Belgium, Germany, Sweden and Poland [7, 11–13]. It has also been recommended that Poland, Germany and Belgium take action regarding the management of populations of this species [6]. In Poland, there are three known sites where *S*. *tomentosa* occurs: the Lower Silesian Wilderness (Bory Dolnośląskie), the Niemodlinski Forests (Bory Niemodlińskie) and Drawski Forests (Puszcza Drawska) [6]. In Poland, this shrub often develops patches covering several dozen hectares, and the expansion of this neophyte has been the cause of significant changes in the natural environment and of impediments to forest management [6, 14].

The results of the recent palynological studies indicate on the importance of the reserch into the pollen variability. In a few studies on representatives of the family Rosaceae (*Crataegus*, *Rosa*, *Rubus*, *Spiraea*) it was established, that the highest variability occurs usually in length of the polar axis (P), equatorial diameter (E), P/E ratio and the length of the ectoaperture (Le) [5, 15–17]. Among the pollen biometric traits, they were usually the most important in the taxonomic diagnosis at the genus, section or species level. In case of the intraspecific, interindividual variability on the example of *Rosa canina*, the authors [16] showed, that in order to well characterise a population of a studied species from the point of view of palynology, the plant material should derive from a possibly numerous number of individuals (shrubs). No distinct statistical relations were found to occur between the morphological traits of pollen grains collected from individual plants growing in the localities with different geographical location. There were similarities among grains collected in regions distant from one another, while those from neighbouring parts of the country could be different [15–17, 34]. Previous pollen morphological data for the Spiraeeae has been presented in a few studies [5, 18–23] whereas palynological data on the *Spiraea* genus is scarce. The most detailed information to date was obtained for 12 *Spiraea* species growing in different regions of Siberia and the Far East [5]. Hebda and Chinnappa [19] described *Spiraea* as part of palynological studies on the pollen morphology of Rosaceae in Canada. Liu et al. [22] analyzed 18 Chinese *Spiraea* species and Jun-Ho et al. [23] studied 38 taxa belonging to all nine genera of the Spiraeeae tribe, including 17 *Spiraea* species. The studies cited revealed that the diagnostic features of the *Spiraea* pollen grains were: exine ornamentation (muri length, width and direction, and perforation diameter), endoaperture diameter and structure, equatorial and polar view, and length of the polar axis).

To date, the pollen morphology and variability of *S*. *tomentosa* has not been studied. Therefore, the aim of this study was to recognize, for the first time, the pollen morphology of *S*. *tomentosa* and to describe the intraspecific and inter-individual variability of this species, basing on the pollen morphology. In the authors’ opinion, due to the invasive nature of the studied species in Europe, any new data on its reproduction may be useful in the fight against the expansion of this species.

## Material and methods

### Palynological analysis

The inflorescences of *S*. *tomentosa* were collected from 30 shrubs (individuals) growing in 30 localities, located in the three centers of occurrence of this species in Poland (Bory Dolnośląskie, Bory Niemodlińskie and Puszcza Drawska, Table 1). The plant material were collected in non-protected commercial forests. No specific permissions were required for these locations. The field studies did not involve any endangered or protected species.

**Table 1:** The list of localities, from which the plant material (*S*. *tomentosa* inflorescences) was collected.

No	Locality	Forest complex	Geographical coordinates N/E		Date	Collector
1	Ruszów	Bory Dolnośląskie	51º20'38"	15º07'08"	31.07.17	Wiatrowska B.
2	Polana	Bory Dolnośląskie	51º37'40"	15º03'07"	31.07.17	Wiatrowska B.
3	Polana	Bory Dolnośląskie	51º25'44"	15º06'22"	31.07.17	Wiatrowska B.
4	Ruszów	Bory Dolnośląskie	51º27'32"	15º07'28"	31.07.17	Wiatrowska B.
5	Ruszów	Bory Dolnośląskie	51º25'44"	15º06'22"	31.07.17	Wiatrowska B.
6	Polana	Bory Dolnośląskie	51º22'01"	15º09'52"	31.07.17	Wiatrowska B.
7	Polana	Bory Dolnośląskie	51º22'03"	15º09'60"	31.07.17	Wiatrowska B.
8	Polana	Bory Dolnośląskie	51º25'43"	15º09'24"	31.07.17	Wiatrowska B.
9	Ruszów	Bory Dolnośląskie	51º24'34"	15º09'36"	31.07.17	Wiatrowska B.
10	Ruszów	Bory Dolnośląskie	51º23'36"	15º10'24"	31.07.17	Wiatrowska B.
11	Gozdnica	Bory Dolnośląskie	51º25'44"	15º06'22"	31.07.17	Wiatrowska B.
12	Ruszów	Bory Dolnośląskie	51º24'50"	15º08'34"	31.07.17	Wiatrowska B.
13	Ruszów	Bory Dolnośląskie	51º24'45"	15º09'25"	31.07.17	Wiatrowska B.
14	Tułowice	Bory Niemodlińskie	50º38'18"	17º38'24"	01.08.17	Wiatrowska B.
15	Tułowice	Bory Niemodlińskie	50º38'19"	17º38'13"	01.08.17	Wiatrowska B.
16	Tułowice	Bory Niemodlińskie	50º37'30"	17º42'40"	01.08.17	Wiatrowska B.
17	Tułowice	Bory Niemodlińskie	50º38'18"	17º38'24"	01.08.17	Wiatrowska B.
18	Tułowice	Bory Niemodlińskie	50º59'30"	17º17'60"	01.08.17	Wiatrowska B.
19	Szydłów	Bory Niemodlińskie	50º37'60"	17º42'04"	01.08.17	Wiatrowska B.
20	Szydłów	Bory Niemodlińskie	50º36'50"	17º35'33"	01.08.17	Wiatrowska B.
21	Tułowice	Bory Niemodlińskie	50º37'46"	17º36'21"	01.08.17	Wiatrowska B.
22	Tułowice	Bory Niemodlińskie	50º59'30"	17º57'51"	02.08.17	Wiatrowska B.
23	Tułowice	Bory Niemodlińskie	50º59'45"	17º60'59"	02.08.17	Wiatrowska B.
24	Tułowice	Bory Niemodlińskie	50º59'55"	17º60'40"	02.08.17	Wiatrowska B.
25	Wygon	Puszcza Drawska	53º00'50"	15º35'16"	10.08.17	Wiatrowska B.
26	Piaseczno	Puszcza Drawska	53º01'20"	15º35'26"	10.08.17	Wiatrowska B.
27	Osowiec	Puszcza Drawska	53º00'05"	15º55'16"	10.08.17	Wiatrowska B.
28	Dobiegniew	Puszcza Drawska	52º57'19"	15°55'54"	10.08.17	Wiatrowska B.
29	Piaseczno	Puszcza Drawska	53º07'16"	15°47'09"	11.08.17	Wiatrowska B.
30	Pestkownica	Puszcza Drawska	53º01'04"	16°03'01"	11.08.17	Wiatrowska B.

Each sample consisted of 30 randomly selected, mature and correctly formed pollen grains. In total, 900 pollen grains were studied. All the samples were acetolysed according to slightly modified Erdtman’s method [24, 25]. The flowers were taken from the bushes and dried in envelopes. For SEM and LM investigations, the dried material (flowers) was placed in test-tubes (10–15 ml). The samples were flooded with iced acetic acid and centrifuged for 4 minutes. Then the material were placed in acetolysis mixture, which consisted of acetic anhydrite (9 portions) and concentrated sulphuric acid (1 portion). The acetolysis process lasted 2.5 minutes. Immediately after acetolysis, the tubes with the mixture were centrifuged for 8 minutes. The mixture was poured out and the samples were filled with 7–8 ml of acetic acid and centrifuged for 5 minutes. Finally prepared material was divided into two parts. One half was immersed in alcohol solution of glycerine (for LM) and the other in ethyl alcohol 96% (for SEM).

In this paper we applied a conventional method, acetolised pollen was measured with use of LM, following other authors who worked on *Spiraea* pollen grains [5, 18–21]. Thus, the measurements obtained in presented paper were comparable to available palinological literature.

Morphological observations were carried out using a light microscope (Biolar 2308, Nikon HFX-DX) and a scanning electron microscope (Hitachi S - 3000N). For SEM observations, pollen was placed on the microscopic tables and sprayed with gold. SEM examination was carried out at the Adam Mickiewicz University in Poznań.

The pollen grains were measured using an eyepiece (ocular) with a scale, and the measurement results were recalculated into micrometers by multiplying them by 2.

Nine quantitative features of the pollen grains were analysed, i.e. the length of the polar axis (P) and equatorial diameter (E), the length of the ectoaperture (Le), the thickness of the exine along the polar axis and equatorial diameter (Exp, Exe), the distance between the apices of two ectocolpi (d) and P/E, Le/P ratios, and the apocolpium index (P.A.I.—d/E ratio). The following qualitative features were also analysed: the outline, shape and exine ornamentation.

The palynological terminology was adopted according to two publications [26, 27].

### Statistical analysis

Firstly, the normality of the distributions of the studied traits (P, E, Exp, Le, d, P/E, Exp/P, Le/P and d/E) was tested using Shapiro-Wilk’s normality test [28]. A multivariate analysis of variance (MANOVA) was performed based on the following model using a MANOVA procedure in GenStat 18: Y = XT+E, where: Y is (*n*×*p*)–the dimensional matrix of observations, *n* is the total number of observations, *p* is the number of traits (in this study *p* = 9), X is (*n*×*k*)–the dimensional matrix of design, *k* is the number of shrubs (in this study *k* = 30), T is (*k*×*p*)–the dimensional matrix of unknown effects, and E–is (*n*×*p*)–the dimensional matrix of residuals. Following this, one-way analyses of variance (ANOVA) were performed in order to verify the null-hypothesis of a lack of shrub effect, opposite of the alternative hypothesis of a significant differences among the shrubs, in terms of the values of the observed traits, independently for each trait, based on the following model: *y*_*ij*_ = *μ*+*τ*_*i*_+*ε*_*ij*_, where: *y*_*ij*_ is *j*th observation of *i*th shrub, *μ* is the grand mean, *τ*_*i*_ is the effect of *i*th shrub and *ε*_*ij*_ is an error observation. The minimal and maximal values of the traits as well as the arithmetic means and coefficients of variation–CV (in %)–were calculated. Moreover, Fisher’s least significant differences (LSDs) were estimated at a significance level of α = 0.001. The relationships between the observed traits were assessed based on Pearson’s correlation coefficients using a FCORRELATION procedure in GenStat 18. The results were also analysed using multivariate methods. A canonical variate analysis was applied in order to present a multi-trait assessment of the similarity of the tested shrubs in a lower number of dimensions with the least possible loss of information [29]. This made it possible to illustrate in graphic form any variation in the shrubs in terms of all the observed traits. Mahalanobis distance was suggested as a measure of “polytrait” shrub similarity [30], the significance of which was verified by means of critical value D_α_ called “the least significant distance” [31]. Mahalanobis distances were calculated for the shrubs. The differences among the analysed shrubs were verified by cluster analysis using the nearest neighbour method and Euclidean distances [32]. All the analyses were conducted using the GenStat 18 statistical software package.

## Results

### Morphological description of pollen

**Fig 1:**
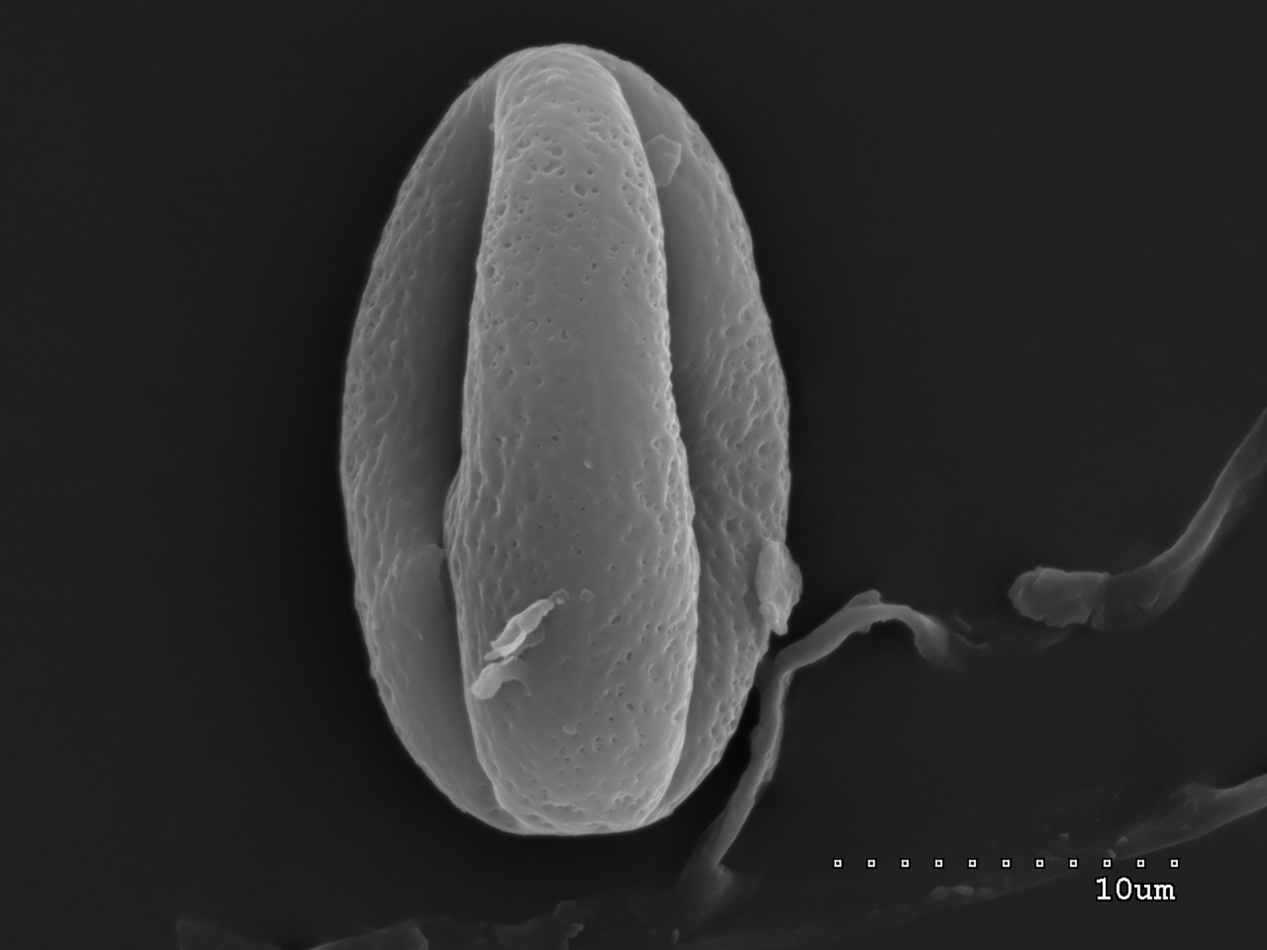
Pollen grain of *S*. *tomentosa* in equatorial view with two colpori.

**Fig 2:**
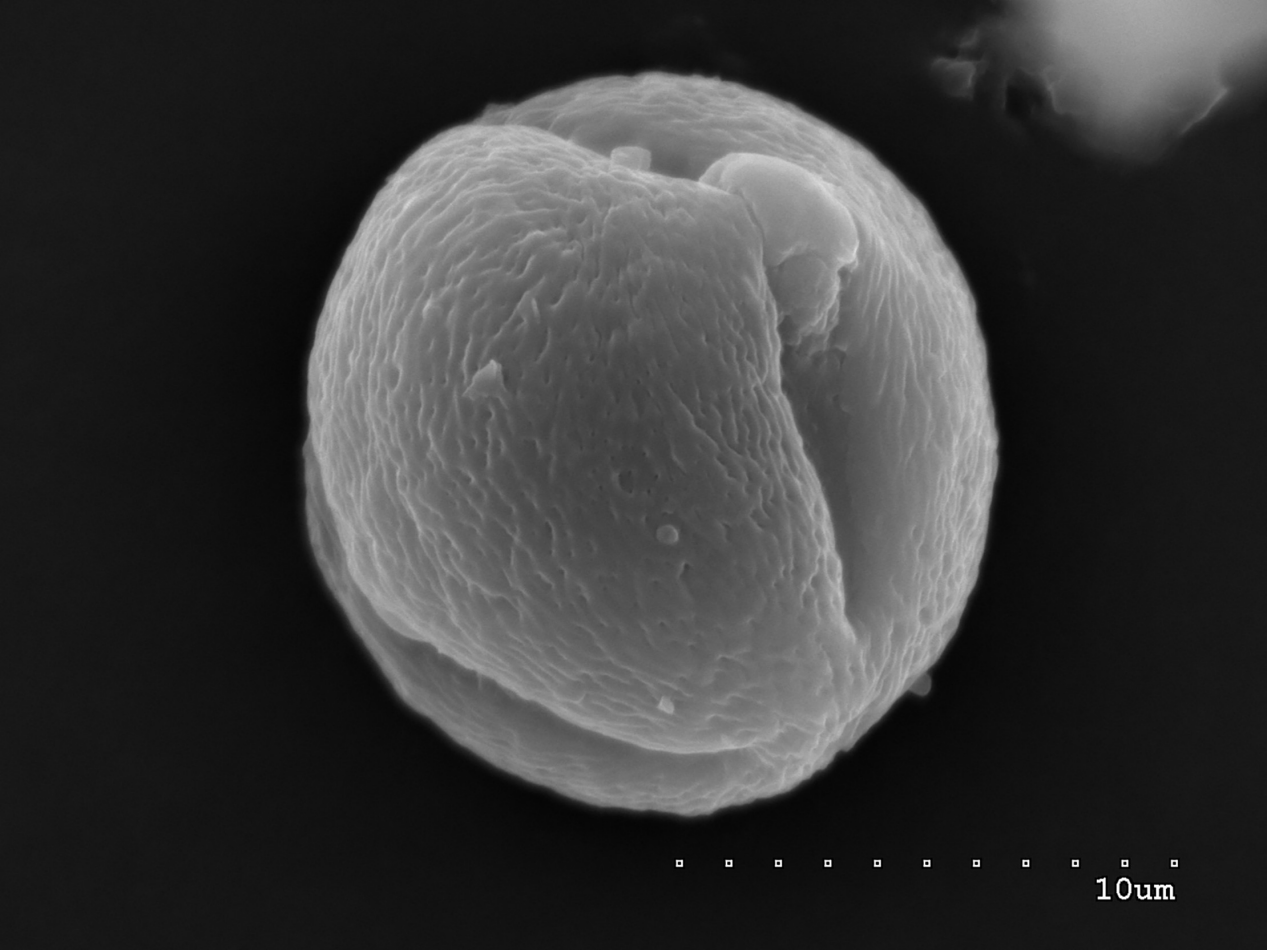
Pollen grain in polar view; convex operculum visible in middle of narrow colporus.

**Fig 3:**
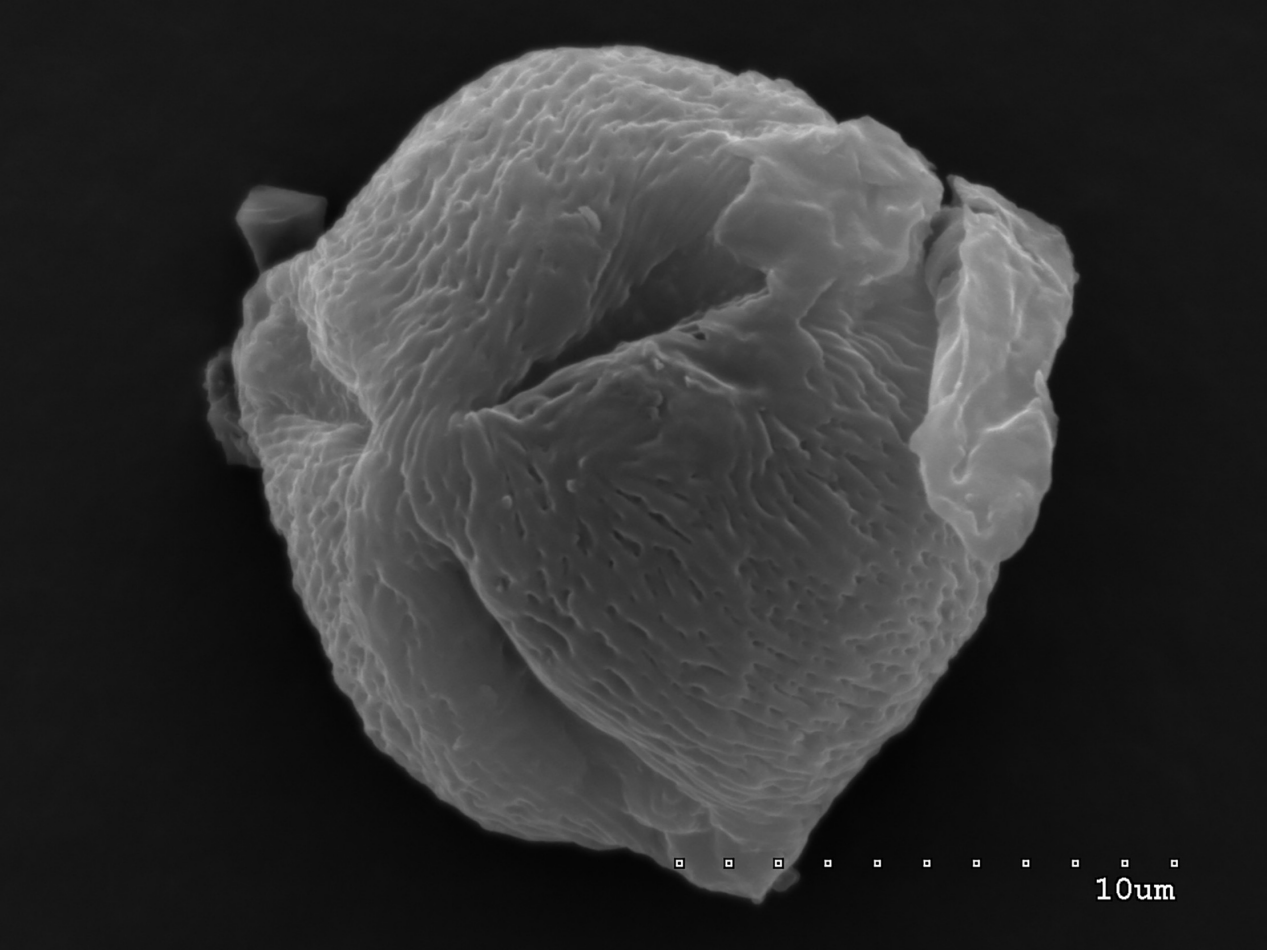
Pollen grain in polar view with three narrow colpori; striate exine ornamentation with striae and muri visible.

**Fig 4:**
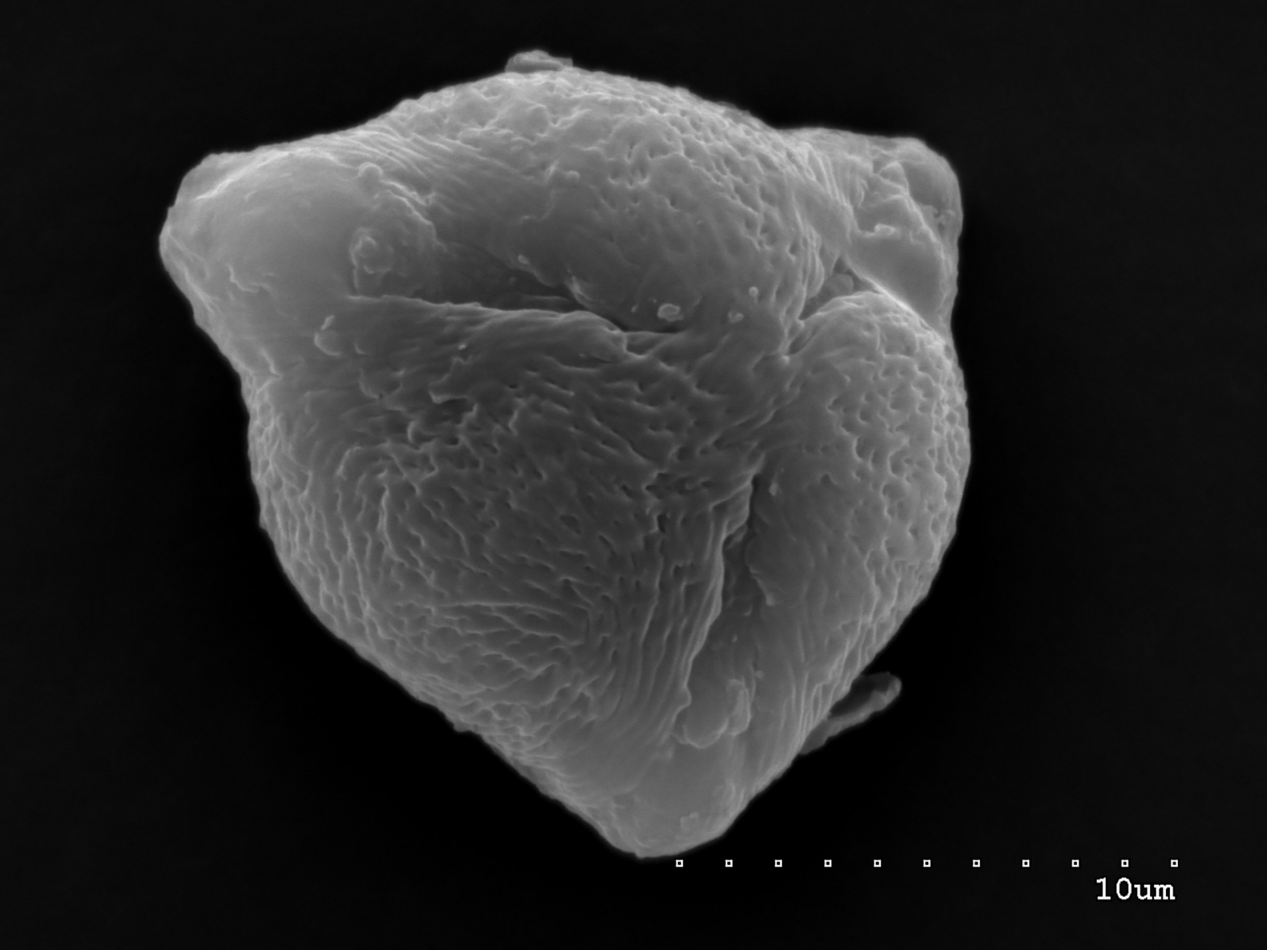
Pollen grain in polar view with three narrow colpori, each one with large, convex operculum.

**Fig 5:**
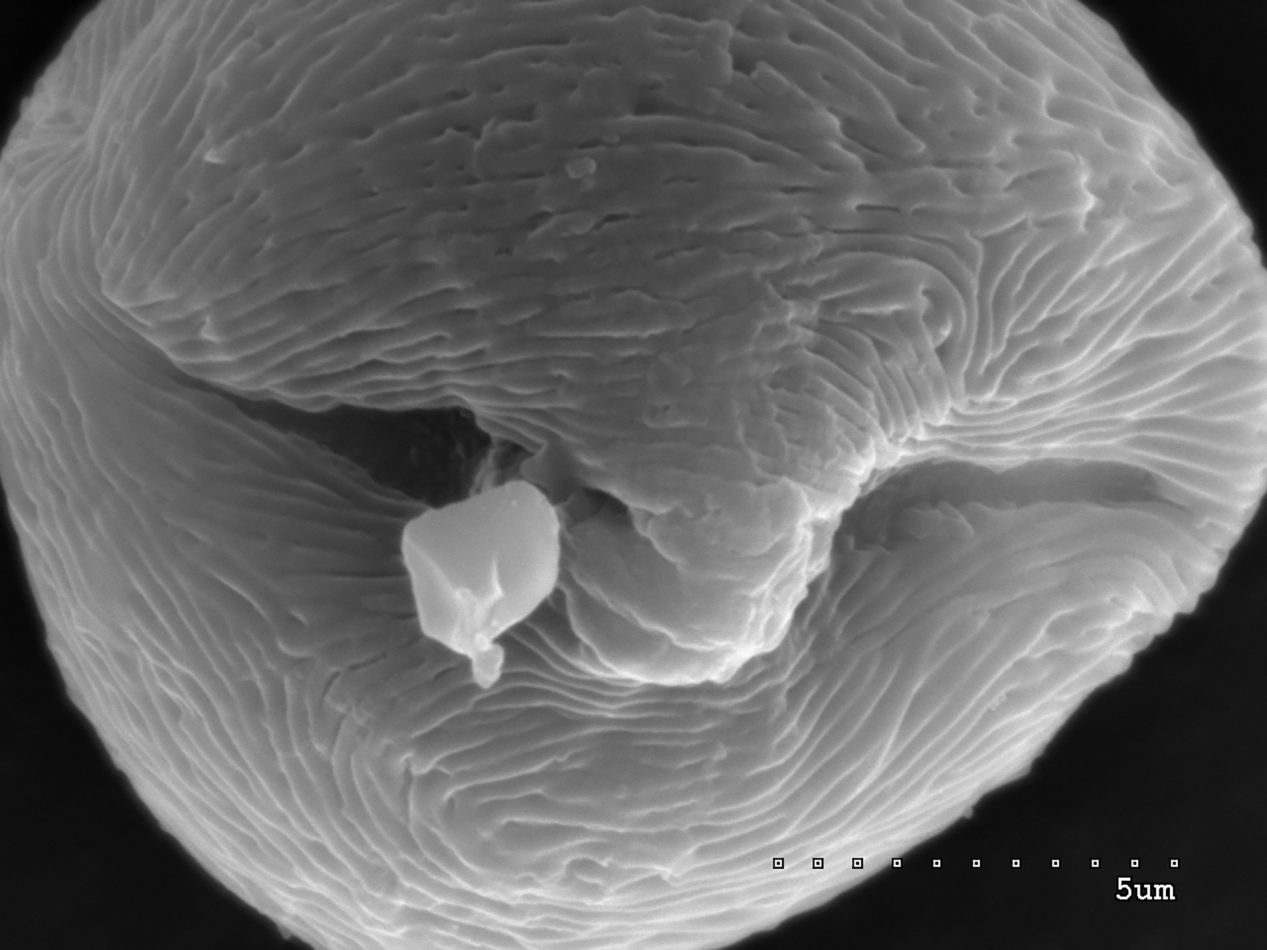
Colporus with operculum; granulate colpus membrane ornamentation visible.

**Fig 6:**
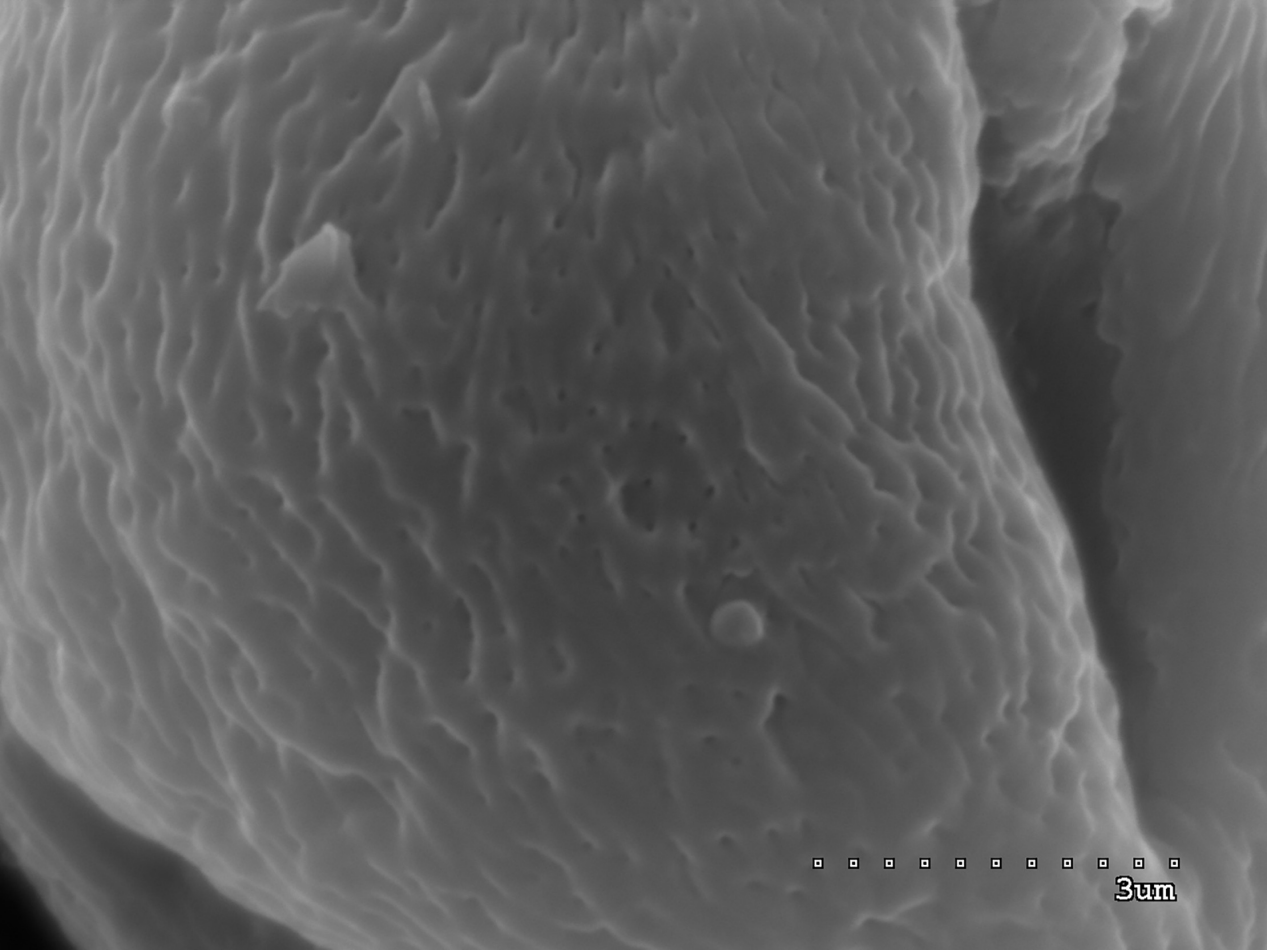
Striate exine ornamentation with small, circular perforations.

A description of the pollen grain morphology of the *S*. *tomentosa* samples studied was given below and illustrated in the SEM photographs (Figs 1–6). The morphological observations for the quantitative features were summarized in Tables 2–4.

**Table 2:** Minimal, maximal and mean values as well as coefficients of variation (CV) for P, E and Exp of shrubs.

Shrub	P			E			Exp		
	Mean	Min-Max	CV	Mean	Min-Max	CV	Mean	Min-Max	CV
1	16.27	12–20	11.06	15.8	14–18	8.38	1.633	1–4	40.95
2	16.33	14–20	10.21	16.2	14–18	8.17	1.667	1–2	28.76
3	16.4	14–20	9.28	16.07	14–18	7.65	1.9	1–4	44.47
4	16	14–18	8.69	15.47	14–18	8.94	1.6	1–3	35.20
5	16.13	14–20	9.17	15.87	12–18	8.06	1.467	1–2	34.59
6	16.27	14–18	7.73	16.33	14–18	7.93	1.833	1–4	35.34
7	15.6	14–18	7.06	16	14–20	8.69	1.4	1–2	35.59
8	16.33	14–20	8.56	16.93	14–20	9.17	1.633	1–2	30.01
9	16.33	14–18	6.50	16.6	14–20	8.46	1.8	1–4	36.91
10	16.53	14–20	7.74	16.6	14–20	8.46	1.633	1–2	30.01
11	15.47	14–18	7.54	16	14–18	7.34	1.567	1–2	32.16
12	15.47	14–18	7.54	16.47	14–18	7.60	1.7	1–4	38.31
13	15.93	14–18	6.15	16.6	14–18	7.85	1.5	1–3	38.16
14	15.53	14–18	7.32	15.67	14–18	6.77	1.367	1–2	35.85
15	15.93	14–18	7.72	15.47	12–18	8.94	1.5	1–2	33.90
16	15.47	12–18	8.94	15.47	12–18	9.56	1.467	1–3	38.94
17	15.87	14–18	7.35	15.47	12–18	7.54	1.433	1–2	35.17
18	15.67	14–18	7.56	15.27	14–18	8.76	1.467	1–2	34.59
19	15.6	14–18	6.21	15.73	14–18	7.27	1.533	1–3	37.27
20	15.53	14–18	8.06	15.67	14–18	5.88	1.4	1–2	35.59
21	16.6	14–20	8.46	15.73	14–18	6.45	1.433	1–2	35.17
22	15.93	14–18	6.98	15.67	14–18	5.88	1.4	1–2	35.59
23	16.53	14–20	7.74	16.07	14–18	6.10	1.567	1–2	32.16
24	15.8	14–18	6.08	16.13	14–18	7.93	1.467	1–2	34.59
25	14.93	12–16	7.66	15.2	14–18	7.41	1.133	1–2	30.51
26	15.73	14–18	8.66	15.6	14–20	10.86	1.633	1–3	34.05
27	15.6	14–18	7.83	16.27	14–18	7.03	1.567	1–2	32.16
28	15.27	14–18	7.28	15.4	14–16	6.05	1.6	1–2	31.14
29	16.13	14–20	8.57	16.27	14–20	8.98	1.533	1–2	33.10
30	16.27	14–18	5.33	16.53	14–18	7.74	1.6	1–2	31.14
LSD_0.01_	1.08			1.08			0.47		
*F* statistic	3.34***			3.90***			2.34***		

P–the length of the polar axis, E–equatorial diameter, Exp–the thickness of the exine along the polar axis, CV–coefficient of variation

*** P<0.001

**Table 3:** Minimal, maximal and mean values as well as coefficients of variation (CV) for Le, d and P/E ratio of shrubs.

Shrub	Le			d			P/E		
	Mean	Min-Max	CV	Mean	Min-Max	CV	Mean	Min-Max	CV
1	13	10–16	13.25	2.7	1–6	43.67	1.04	0.86–1.43	15.05
2	13.2	10–18	12.95	3.067	2–6	37.27	1.02	0.78–1.29	13.60
3	12.67	10–16	13.32	2.733	1–6	47.97	1.03	0.78–1.29	13.13
4	12.87	12–16	8.83	3.1	1–6	41.81	1.04	0.88–1.29	11.52
5	13.2	10–16	10.22	3.133	1–6	37.25	1.02	0.88–1.50	13.37
6	13.4	10–16	10.48	3.067	2–6	44.44	1.00	0.78–1.14	9.35
7	12.6	10–16	11.90	2.833	1–6	44.55	0.98	0.80–1.29	11.02
8	13.53	10–18	12.69	2.733	2–6	40.69	0.97	0.78–1.43	12.54
9	13	12–14	7.82	3.133	1–6	44.14	0.99	0.78–1.29	11.35
10	13.4	10–16	10.48	2.967	1–4	31.28	1.00	0.80–1.25	10.08
11	12.6	10–16	11.90	2.667	1–5	37.27	0.97	0.88–1.14	8.91
12	12.47	10–14	10.89	2.633	1–4	33.80	0.94	0.88–1.14	8.84
13	12.87	10–16	11.31	3	1–5	30.33	0.97	0.88–1.14	9.14
14	12.67	10–16	11.97	2.733	1–4	34.54	1.00	0.78–1.29	11.33
15	12.93	10–16	10.54	2.733	2–4	27.08	1.04	0.88–1.17	8.64
16	12.4	10–16	9.85	2.7	1–4	32.48	1.01	0.75–1.50	13.03
17	13	10–16	10.50	2.733	2–4	31.76	1.03	0.88–1.33	10.33
18	12.47	10–14	11.68	2.633	1–4	40.49	1.03	0.88–1.14	7.21
19	12.53	10–16	11.04	2.667	1–4	34.57	1.00	0.88–1.29	8.46
20	12.73	10–16	10.50	2.8	1–4	30.25	0.99	0.88–1.14	6.69
21	13.67	12–16	10.23	2.6	1–4	38.58	1.06	0.88–1.29	8.47
22	13.13	10–16	11.09	2.3	1–4	39.78	1.02	0.88–1.14	7.39
23	13.33	12–18	10.67	2.5	1–4	40.36	1.03	0.88–1.29	10.08
24	12.67	10–16	11.22	2.433	1–4	42.75	0.98	0.88–1.14	7.59
25	12.27	10–14	10.25	2.4	1–4	37.25	0.99	0.86–1.14	9.20
26	12.27	10–16	12.65	2.367	1–4	45.04	1.02	0.80–1.29	11.40
27	12.33	10–16	12.11	2.667	1–4	35.96	0.96	0.78–1.14	9.10
28	11.93	10–14	6.94	2.5	1–4	38.96	0.99	0.88–1.14	8.47
29	12.93	10–18	13.91	2.433	1–4	39.91	1.00	0.78–1.14	9.79
30	12.73	12–14	7.70	2.433	1–4	41.35	0.99	0.88–1.14	7.20
LSD_0.01_	1.21			0.89			0.09		
*F* statistic	5.17***			1.58*			2.05***		

Le–the length of the ectoaperture, d–the distance between the apices of two ectocolpi, P–the length of the polar axis, E–equatorial diameter, CV–coefficient of variation

* P<0.05

*** P<0.001

**Table 4:** Minimal, maximal and mean values as well as coefficients of variation (CV) for Exp/P, Le/P and d/E ratios of shrubs.

Shrub	Exp/P			Le/P			d/E		
	Mean	Min-Max	CV	Mean	Min-Max	CV	Mean	Min-Max	CV
1	0.100	0.050–0.200	36.75	0.801	0.625–0.889	9.48	0.170	0.063–0.333	41.31
2	0.103	0.056–0.143	31.24	0.810	0.625–0.900	10.44	0.190	0.111–0.375	37.90
3	0.115	0.056–0.250	40.26	0.774	0.600–0.889	11.13	0.169	0.063–0.333	45.17
4	0.099	0.063–0.188	33.35	0.808	0.667–0.889	9.45	0.202	0.063–0.429	44.32
5	0.091	0.056–0.143	33.77	0.820	0.667–0.889	7.73	0.197	0.063–0.333	34.46
6	0.113	0.056–0.222	33.80	0.824	0.667–0.889	7.98	0.187	0.111–0.333	41.95
7	0.090	0.056–0.143	35.64	0.808	0.625–0.889	9.71	0.177	0.063–0.429	45.02
8	0.100	0.050–0.143	29.58	0.829	0.714–1.000	10.15	0.162	0.100–0.333	39.17
9	0.110	0.056–0.222	33.98	0.797	0.667–0.875	7.64	0.189	0.056–0.375	43.39
10	0.099	0.056–0.143	30.90	0.811	0.667–0.889	8.19	0.179	0.063–0.286	31.73
11	0.101	0.063–0.143	31.36	0.815	0.625–0.889	9.44	0.169	0.063–0.313	39.99
12	0.110	0.056–0.222	35.55	0.807	0.625–0.875	9.38	0.161	0.063–0.286	34.62
13	0.094	0.056–0.188	38.43	0.807	0.714–0.889	8.14	0.181	0.063–0.286	30.11
14	0.088	0.056–0.143	34.61	0.814	0.556–1.000	11.24	0.175	0.063–0.286	34.62
15	0.094	0.056–0.143	32.01	0.812	0.714–0.889	7.66	0.177	0.111–0.250	24.93
16	0.094	0.063–0.167	35.68	0.804	0.667–0.889	7.95	0.174	0.063–0.286	30.07
17	0.091	0.056–0.143	36.87	0.820	0.667–0.889	8.17	0.177	0.111–0.286	30.74
18	0.094	0.056–0.143	35.45	0.795	0.714–0.875	8.19	0.174	0.063–0.286	42.54
19	0.098	0.063–0.188	36.11	0.803	0.625–0.889	8.95	0.170	0.063–0.286	35.22
20	0.089	0.063–0.143	32.08	0.820	0.714–0.889	7.44	0.180	0.063–0.286	31.44
21	0.087	0.056–0.143	36.01	0.824	0.750–0.889	6.96	0.166	0.063–0.286	38.95
22	0.088	0.056–0.143	34.97	0.824	0.714–0.889	7.63	0.147	0.056–0.250	40.20
23	0.095	0.050–0.125	31.89	0.807	0.667–0.900	8.00	0.156	0.056–0.250	39.25
24	0.093	0.063–0.143	34.28	0.801	0.625–0.889	8.97	0.153	0.056–0.286	46.12
25	0.076	0.063–0.143	29.11	0.822	0.714–0.875	7.40	0.157	0.071–0.250	34.94
26	0.105	0.056–0.214	37.33	0.780	0.625–0.889	9.17	0.153	0.056–0.286	45.92
27	0.100	0.063–0.143	31.65	0.790	0.714–0.889	8.02	0.164	0.056–0.250	35.60
28	0.104	0.063–0.143	28.50	0.784	0.714–0.875	7.09	0.163	0.063–0.286	38.53
29	0.095	0.056–0.143	32.43	0.800	0.625–0.900	8.96	0.151	0.056–0.286	40.05
30	0.098	0.056–0.125	30.49	0.784	0.667–0.875	7.38	0.147	0.056–0.250	39.30
LSD_0.01_	0.03			0.06			0.06		
*F* statistic	1.93**			1.24			1.44		

Exp–the thickness of the exine along the polar axis, P–the length of the polar axis, Le–the length of the ectoaperture, d–the distance between the apices of two ectocolpi, E–equatorial diameter, CV–coefficient of variation

** P<0.01

The pollen grains of the studied species occur as radially symmetric, tricolporate, isopolar monads (Figs 1–4).

According to Erdtman’s [24] pollen size classification, all of the analyzed pollen grains were small (10.1–25 μm). The length of the polar axis (P) was 15.92 (12.00–20.00) μm. The smallest P (12.00 μm) were found in pollen samples 1.16 and 25, and the largest ones (20.00 μm) in many samples, which indicates that many grains reach high values of this feature. Most of the pollen grains had a similar length (P); the average value of this feature ranged from 14.93 to 16.60 μm.

The mean length of the equatorial diameter (E) was 15.95 (12.00–20.00) μm. The smallest value of this feature (12.00 μm) was found in the pollen from samples 5 and 15–17, and the largest ones (20.00 μm) in pollen from eight samples. The average value of the equatorial diameter varied between 15.20 and 16.93 μm.

The polar area index (PAI) or apocolpium index (d/E ratio) averaged 0.17 (0.06–0.43). The lowest mean values of this ratio (0.06) were recorded in many samples, which shows that many grains reach the lowest distance between the apices of two ectocolpi (d), while the highest (0.43) occurred in samples 4 and 7. The average values of this trait (0.15–0.20) indicated that it was similar in all the studied pollen grains.

The outline in polar view was mostly circular or triangular with obtuse apices, more rarely elliptic, and in equatorial view, it was circular or elliptic (Figs 1–4).

The mean P/E ratio was 1.00, and ranged from 0.75 in sample 16 to 1.50 in sample 5. The shape of the examined pollen grains was most frequently spheroidal (465 grains—51.7%), rarely oblate-spheroidal (236–26.2%) or prolate-spheroidal (168–18.7%) and very rarely subprolate (25–2.8%) or prolate (6–0.7%).

The mean exine thickness was 1.55 μm (with a significant range of 1.00–4.00 μm). The average value of this feature was also relatively high (1.13–1.90 μm). The relative thickness of the exine (Exp/P ratio) averaged 0.10 (0.05–0.25).

The exine ornamentation was striate (Figs 1–6). The striae and muri usually ran parallel to the polar axis but they also frequently formed loops. They were straight or forked and of varying length and width (Figs 2–5). Either the muri were wider than the striae or the widths of the muri and striae were similar and averaged from 0.1 to 0.3 μm. Small, circular or elliptic perforations of similar diameters (on average—0.1μm) were found at the bottom of the striae (Fig 6).

Pollen grains usually possess three apertures—colpori. The colpi were distributed symmetrically, elongated, and narrowed toward the poles, with granular aperture membranes. Their mean length varied between 10.00 and 18.00 μm, with an average of 12.83 μm. Usually, the length of the colpi constituted 81% of the polar axis length and ranged from 56 to 100%. Their width was variable and usually the greatest in the equatorial region.

Frequently, the colpus membrane was partially covered by an operculum (a coherent exine structure covering an aperture). The operculum was usually situated in the central part of the ectocolpus; sometimes it was placed asymmetrically and partially covering the ectocolpus. The operculum sculpture was psilate. Its surface was usually corrugated. The pollen grains of *S*. *tomentosa* had large convex operculum (Figs 2–5).

The endoapertures were usually located in the middle of the colpi, less frequently asymmetrically, usually singly. They were circular or elliptic in outline with irregular margins.

### Intraspecific and interindividual variability of pollen grains

The results of the MANOVA performed indicated that all the samples were significantly different with regard to all of the nine quantitative traits (Wilk’s λ = 0.6005; F_29,870_ = 1.74; *P*<0.0001). The results of ANOVA indicated that the main effects of the shrubs were significant for seven observed traits: P, E, Exp, Le, d, P/E and Exp/P (Tables 2–4). The differences between the shrubs for Le/P and d/E were not statistically significant (Table 4). The mean values and coefficients of variation (CV) for the observed traits indicated a high variability among the tested samples for which significant differences were found in terms of all the analysed morphological traits (Tables 2–4).

The correlation analysis performed indicated statistically significant correlation coefficients for 15 out of 36 coefficients (Table 5). Twelve out of 15 significantly correlated pairs of traits were characterized by positive correlation coefficients. Negative correlation coefficients were observed between: P/E and E (-0.525), Le/P and Exp (-0.428), and Le/P and Exp/P (-0.489). In the case of 21 pairs of traits, no significant correlation was established. This means that a value increase in one trait in a given pair led to a value increase in the second trait.

**Table 5:** Correlation coefficients between observed features.

Trait	P	E	Exp	Le	d	P/E	Exp/P	Le/P	d/E
P	1								
E	0.52**	1							
Exp	0.54**	0.55**	1						
Le	0.83***	0.43*	0.19	1					
d	0.32	0.29	0.36	0.38*	1				
P/E	0.45*	-0.53**	-0.02	0.37*	0.05	1			
Exp/P	0.32	0.47**	0.97***	-0.02	0.30	-0.15	1		
Le/P	0.04	-0.01	-0.43*	0.58***	0.24	0.03	-0.49**	1	
d/E	0.15	-0.04	0.19	0.25	0.94***	0.22	0.16	0.30	1

P–the length of the polar axis, E–equatorial diameter, Le–the length of the ectoaperture, Exp–the thickness of the exine along the polar axis, Exe–the thickness of the exine along the equatorial diameter, d–the distance between the apices of two ectocolpi

* P<0.05

** P<0.01

*** P<0.001

**Fig 7:**
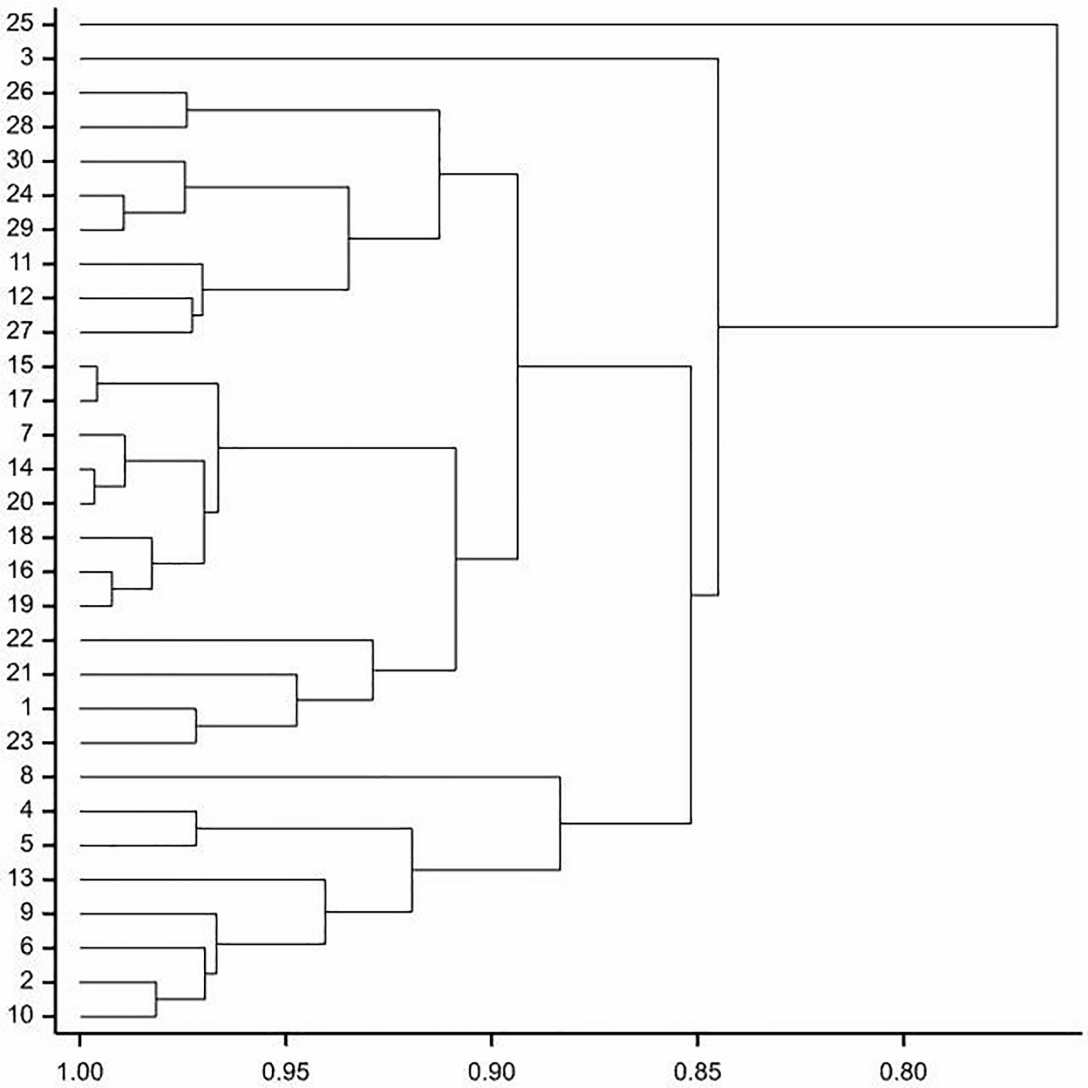
Dendrogram of cluster groupings of *S*. *tomentosa* shrubs based on all nine morphological traits.

In the presented dendrogram, as a result of agglomeration grouping using the Ward method, all the examined *S*. *tomentosa* samples were divided into two groups (Fig 7). The first group (I) comprised one sample, number 25, collected from Wygon in Puszcza Drawska. The second one (II) included all the other samples and was divided into two subgroups: II A—sample 3 and II B—all other samples. The subgroup II B was divided into three subgroups: II B1 (2, 4–6, 8–10, 13), II B2 (1, 7, 15–23) and II B3 which included all the other samples.

Dendrogram indicates, that there was no clear dependencies between the pollen traits and the locality from which the samples were collected (e.g. samples collected from the Polana—2, 3, and 6–8, have different pollen grains and the ones from Tułowice—14–18, were usually very similar) (Fig 7).

**Fig 8:**
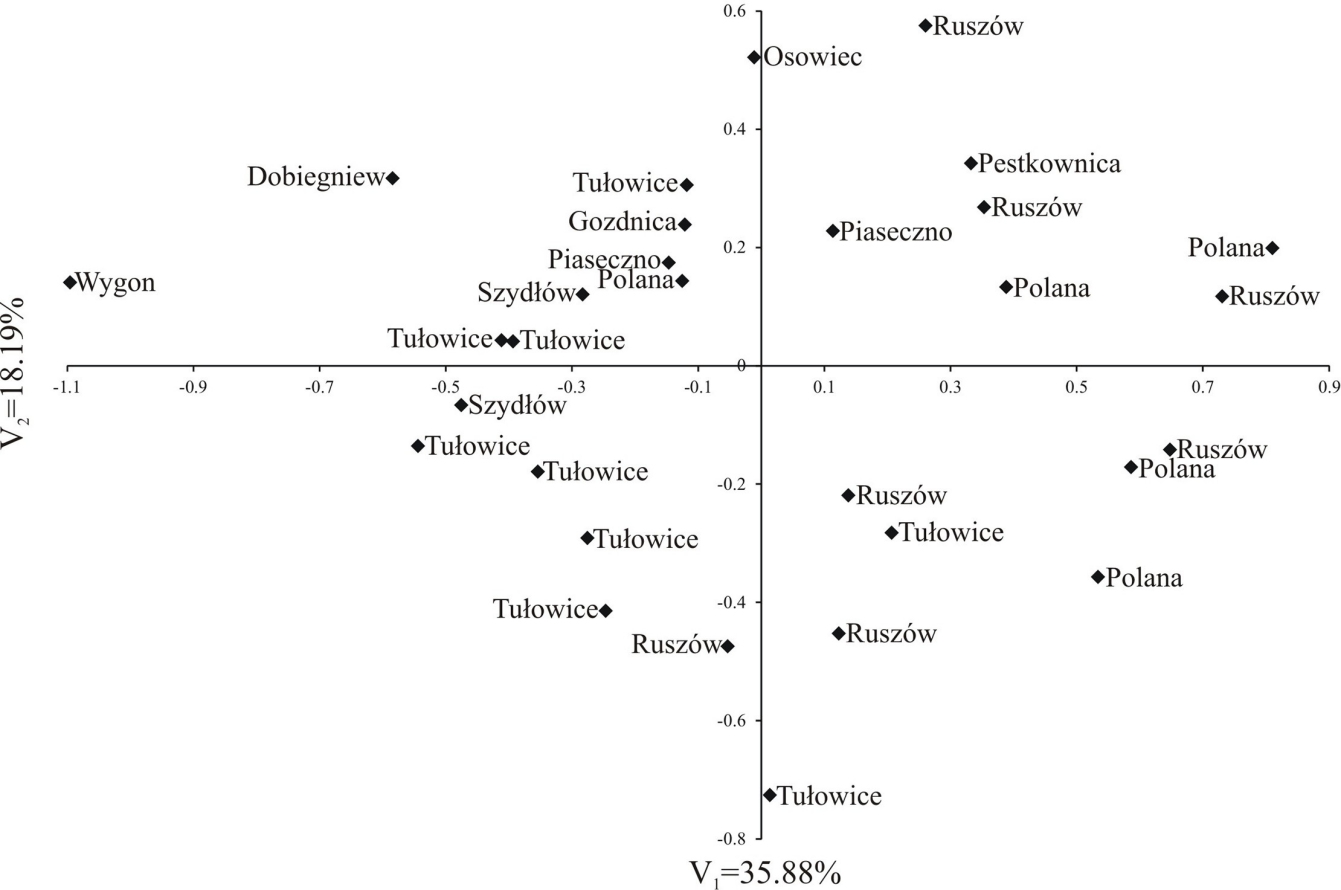
Distribution of 30 *S*. *tomentosa* shrubs in space of two first canonical variables.

Fig 8 shows the variability of the pollen grain features of the 30 studied *S*. *tomentosa* individuals (shrubs) in terms of the first two canonical variables. In the graph, the coordinates of the point for particular shrubs are the values for the first and second canonical variables, respectively. The first two canonical variables accounted for 54.07% of the total multivariate variability between the individual shrubs. Significant positive linear relationships with the first canonical variables were found for the P, E, Exp, Le, d and Exp/P (Table 6). The second canonical variable was significantly positively correlated with E and negatively with P, Le, P/E, Le/P and d/E (Table 6). The goal of the study was to establish whether pollen grains collected from various steeplebush shrubs growing in different habitat conditions (soil and climate) would differ from one another. Four groups of shrubs were distinguished. The majority of the examined individuals were found in the first group (I). Just one to three individuals fell into the other three groups—II—Wygon, III—Ruszów, Osowiec, Dobiegniew and IV—Tułowice (Fig 8).

**Table 6:** Correlation coefficients between first two canonical variables and original traits.

Trait	First canonical variable	Second canonical variable
P	0.790***	-0.471**
E	0.875***	0.375*
Exp	0.760***	0.136
Le	0.625***	-0.632***
d	0.498**	-0.341
P/E	-0.115	-0.859***
Exp/P	0.636***	0.283
Le/P	-0.028	-0.469**
d/E	0.22	-0.481**
Percentage of explained multivariate variability	35.88%	18.19%

P–the length of the polar axis, E–equatorial diameter, Le–the length of the ectoaperture, Exp–the thickness of the exine along the polar axis, Exe–the thickness of the exine along the equatorial diameter, d–the distance between the apices of two ectocolpi

* P<0.05

** P<0.01

*** P<0.001

The analysis of the localities (Fig 8) from which the flowers (pollen grains) from the individual *S*. *tomentosa* shrubs were collected showed that the largest group I, contained almost all the analysed pollen grains samples, coming from the same localities [e.g. Polana (samples 1 and 6–8), Tułowice (14–18), Piaseczno (26–29) and many others], as well as from places geographically distant from one another [e.g. from Ruszów (9), from Szydłów (20) or Pestkownica (30)].

The greatest variation in terms of all the traits, based on the measured Mahalanobis distances, was found for shrubs 8 and 25 (the Mahalanobis distance between them amounted to 2.02). The greatest similarity was found for shrubs 24 and 29 (0.40). The values of the Mahalanobis distances for all pairs of treatments are presented in Table 7.

**Table 7:** Mahalanobis distances between analysed *S*. *tomentosa* shrubs (individuals).

Shrub	1	2	3	4	5	6	7	8	9	10	11	12	13	14	15	16	17	18	19	20	21	22	23	24	25	26	27	28	29
**2**	0.76																												
**3**	0.73	1.19																											
**4**	0.90	0.91	1.27																										
**5**	0.58	0.69	1.16	0.84																									
**6**	0.84	0.83	0.97	1.19	0.88																								
**7**	0.71	0.97	1.17	1.08	0.67	1.10																							
**8**	1.06	0.95	1.27	1.44	1.13	0.93	1.08																						
**9**	0.81	0.87	0.72	1.23	0.92	0.66	1.01	0.80																					
**10**	0.73	0.57	1.03	1.19	0.76	0.69	0.92	0.67	0.65																				
**11**	0.91	1.00	1.21	0.95	1.01	1.03	0.70	1.11	1.14	1.02																			
**12**	1.05	1.03	1.19	1.30	1.22	1.04	0.86	0.91	0.99	1.01	0.57																		
**13**	0.87	0.90	1.16	1.28	0.83	0.94	0.56	0.74	0.76	0.62	0.86	0.80																	
**14**	0.81	1.20	1.28	1.06	0.80	1.20	0.43	1.31	1.25	1.13	0.70	1.07	0.84																
**15**	0.52	1.00	1.07	0.82	0.62	1.02	0.66	1.31	1.15	1.01	0.79	1.17	0.99	0.47															
**16**	0.70	1.15	1.12	0.90	0.81	1.25	0.53	1.37	1.19	1.23	0.76	1.04	1.01	0.51	0.56														
**17**	0.66	0.84	1.33	0.83	0.59	1.12	0.69	1.32	1.29	1.02	0.83	1.17	1.03	0.64	0.44	0.68													
**18**	0.93	1.22	1.39	0.99	1.04	1.40	0.85	1.60	1.52	1.30	0.81	1.24	1.18	0.63	0.58	0.77	0.63												
**19**	0.71	1.05	1.07	0.95	0.87	1.05	0.55	1.25	1.13	1.02	0.43	0.83	0.84	0.42	0.48	0.56	0.66	0.53											
**20**	0.98	1.29	1.31	0.99	0.96	1.20	0.82	1.50	1.36	1.23	0.68	1.18	1.10	0.58	0.62	0.78	0.83	0.76	0.52										
**21**	0.90	0.98	1.46	1.19	0.91	1.23	1.17	1.43	1.47	0.97	1.22	1.53	1.28	1.13	0.85	1.29	0.74	1.02	1.07	1.12									
**22**	0.81	1.20	1.30	1.22	0.98	1.22	0.85	1.36	1.41	1.10	0.84	1.21	1.12	0.68	0.58	0.89	0.66	0.73	0.63	0.71	0.72								
**23**	0.58	0.84	1.01	1.17	0.86	0.98	0.95	1.10	1.07	0.66	1.00	1.17	0.98	1.01	0.77	1.10	0.80	1.01	0.86	1.04	0.55	0.65							
**24**	0.94	1.14	1.24	1.16	1.14	1.27	0.76	1.11	1.25	1.00	0.55	0.82	0.83	0.75	0.86	0.92	0.94	0.78	0.55	0.83	1.13	0.75	0.86						
**25**	1.43	1.92	1.76	1.63	1.44	1.88	1.11	2.02	1.90	1.88	1.33	1.67	1.60	0.92	1.12	0.93	1.26	1.22	1.11	0.97	1.68	1.11	1.60	1.38					
**26**	0.93	1.10	1.33	1.31	1.22	1.43	0.93	1.42	1.41	1.24	0.96	1.01	1.14	1.02	1.00	0.96	0.88	0.81	0.85	1.32	1.25	1.04	1.04	0.91	1.59				
**27**	0.90	1.14	1.06	1.34	1.09	1.12	0.58	1.10	1.01	0.95	0.66	0.66	0.59	0.72	0.92	0.87	1.04	0.94	0.56	0.94	1.35	0.95	0.98	0.60	1.36	0.88			
**28**	1.01	1.42	1.17	1.23	1.26	1.40	0.90	1.68	1.44	1.44	0.78	1.06	1.26	0.79	0.83	0.71	1.00	0.72	0.54	0.78	1.42	0.91	1.18	0.90	1.03	0.93	0.79		
**29**	0.76	1.04	1.05	1.26	1.03	1.09	0.74	0.92	1.05	0.76	0.75	0.86	0.69	0.79	0.82	0.98	0.94	0.90	0.65	0.98	1.01	0.72	0.64	0.40	1.49	0.90	0.56	1.03	
**30**	0.85	1.05	0.93	1.38	1.18	1.20	0.93	0.99	0.97	0.79	0.97	0.90	0.83	1.14	1.12	1.16	1.19	1.23	0.93	1.22	1.20	1.02	0.71	0.70	1.68	1.03	0.72	1.16	0.54

## Discussion

In the opinion of Liu et al. [22], pollen morphology could provide important evidence for interspecific classification in the *Spiraea* L. genus. Results from previous studies revealed that the diagnostic features of the pollen grains from the *Spiraea* genus were the ora (endoaperture) structure and exine ornamentation (e.g. striae size) [5], striae course, porus diameter, lirae (muri) width, length of polar axis (P), L/E ratio [22] length and direction of the ridge patterns - muri [23] and perforation number and diameter [19, 23].

All of analyzed *S*. *tomentosa* pollen grains were small. In the papers cited previously, the pollen grains of other *Spiraea* species were described most often as small-sized [5, 22, 23] and rarely as medium-sized [5, 23].

Researchers differ in their diagnosis of the pollen shape (P/E ratio) in the *Spiraea* species. The research presented here indicated that the pollen shape of *S*. *tomentosa* was most frequently spheroidal, rarely oblate-spheroidal and prolate-spheroidal, and very rarely subprolate or prolate. These results confirm those found by Jun-Ho et al. [23], who claimed that the pollen shape was from oblate to prolate. However, both these sets of results differ from previous research which only showed a prolate and subspheroidal [22] or elongated spheroidal pollen shape [5]. The reason for these differences may be a simplified description of the pollen shape in the last two publications.

Polyakova and Gataulina [5] showed that, based on aperture (colpori) morphology, it was possible to distinguish two types of pollen grains. The first group of species (*S*. *humilis*, *S*. *salicifolia*, and *S*. *hypericifolia*) had wide ora—endoapertures and in the second group (*S*. *crenata*, *S*. *trilobata*, *S*. *betulifolia*, *S*. *media*, *S*. *alpine*), the endoapertures were narrow. The next important trait was the operculum shape. Jun-Ho et al. [23] observed various types of operculum appearing in the *Spiraea* species. The pollen grains of *S*. *tomentosa* had large, convex, psilate operculum usually situated in the central part of the colporus.

The results presented here concerning the exine ornamentation in species of the *Spiraea* genus were partly confirmed by recent studies by Hebda and Chinnappa [19], Polyakova and Gataulina [5] and Liu et al. [22], who described exine ornamentation as striate or striate-granular. In *S*. *tomentosa* pollen grains, only striate exine ornamentation occurs. All palynologists quoted, as well as the authors of this paper, that the striae and muri course and dimensions were the most important pollen grain features. Such a result was confirmed by Polyakova and Gataulina [5], who reported that in some *Spiraea* species, the striae were crossing and branching, mainly meridionally and in different directions. Sometimes the general direction of the striae changed from meridional to equatorial. The striae were often long, but could also be short. As a rule, they lay tightly and sometimes, on the apocolpium, less tightly. Liu et al. [22] claim that in the *Spiraea* species, exine ornamentation was “fringe and stripe reticular”. These authors described a “special brain-stripe structure in germinal furrow”in *S*. *elegans*. Perhaps the operculum or bridge was being referred to, but palynological terminology was not used, therefore it is difficult to determine what structure they meant. Jun-Ho et al. [23] recognized that all Spiraeae taxa have a striate exine ornamentation pattern, with supratectal ridges (muri) separated by valleys (striae). Although a continuous variation was observed in the exine ornamentation, four types of striate sexine ornamentation could be recognised. The first three types could each be divided into two subtypes based on the diameter of the perforations. In Hebda and Chinnappa’s [19] opinion, in the *Spiraea* genus, perforations were large and often extending onto tectal ridges (muri). Jun-Ho et al. [23] found as large as small perforations in the *Spiraea* species. In *S*. *tomentosa* they were small, circular or elliptic and had similar diameters (on average—0.1μm). In some Spiraeae taxa, without the *Spiraea* genus, Jun-Ho et al. [23] reported Ubisch bodies (orbicules) which in *S*. *tomentosa* were not found.

For the first time, the intraspecific and interindividual variability of *S*. *tomentosa* pollen grains were studied based on nine quantitative features. Statistical analysis of the studied traits indicated a high variability among the tested samples. The most variable biometric traits were P, E and Le, while lower variability occurred in P/E, Le/P and d/E ratios (Table 2). In a previous paper [33] for the five *Quercus* taxa, similar results were obtained for P, E and Le, but most of the other features were more variable. Almost half (16) of the 36 studied pairs of features were characterized by positive correlation coefficients. In the case of the *Quercus* taxa, the correlation analysis performed indicated statistically significant correlation coefficients in 29 out of 36 studied trait pairs [33]. These results were probably related to the larger, intra- and interspecific differences between the studied *Quercus* taxa, compared to the variability of one species—*S*. *tomentosa*.

The pollen grains from samples 25, 21, 27, 28 and 12 were the most different from all the other analysed samples collected from 30 *S*. *tomentosa* shrubs. The analysis of the sites of the studied samples (pollen grains from particular *Spiraea* shrubs) showed that in individual groups, the shrubs examined derived from the same sites as well as from places geographically distant from one another. Similar results were obtained by Wrońska-Pilarek et al. [33–35] in the palynological studies of selected species from the *Crataegus* and *Quercus* genera.

In conclusion, the results of the study presented here showed that the analysed morphological features of the pollen grains from 30 samples, collected from 30 *S*. *tomentosa* shrubs, did not allow the isolation of individual samples, but only their groups. In the opinion of the authors, the pollen morphology was not the source of diagnostic, taxonomic characters at species level. However, the pollen morphology could provide important evidence for interspecific classification [22] and may have potential taxonomic value at the sectional level [23].
